# Nickel–cobalt bimetallic sulfide NiCo_2_S_4_ nanostructures for a robust hydrogen evolution reaction in acidic media†

**DOI:** 10.1039/d0ra03191g

**Published:** 2020-06-15

**Authors:** Umair Aftab, Aneela Tahira, Raffaello Mazzaro, Vittorio Morandi, Muhammad Ishaq Abro, Muhammad Moazam Baloch, Cong Yu, Zafar Hussain Ibupoto

**Affiliations:** Mehran University of Engineering and Technology 7680 Jamshoro Sindh Pakistan umair.aftab@faculty.muet.edu.pk; Department of Science and Technology, Campus Norrkoping, Linkoping University SE-60174 Norrkoping Sweden; Institute for Microelectronics and Microsystems, Italian National Research Council, Section of Bologna Via Piero Gobetti 101 40129 Bologna Italy; State Key Laboratory of Electroanalytical Chemistry, Changchun Institute of Applied Chemistry, Chinese Academy of Sciences Changchun People's Republic of China; Dr M. A. Kazi Institute of Chemistry, University of Sindh Jamshoro 76080 Sindh Pakistan zaffar.ibhupoto@usindh.edu.pk

## Abstract

There are many challenges associated with the fabrication of efficient, inexpensive, durable and very stable nonprecious metal catalysts for the hydrogen evolution reaction (HER). In this study, we have designed a facile strategy by tailoring the concentration of precursors to successfully obtain nickel–cobalt bimetallic sulfide (NiCo_2_S_4_) using a simple hydrothermal method. The morphology of the newly prepared NiCo_2_S_4_ comprised a mixture of microparticles and nanorods, which were few microns in dimension. The crystallinity of the composite sample was found to be excellent with a cubic phase. The sample that contained a higher amount of cobalt compared to nickel and produced single-phase NiCo_2_S_4_ exhibited considerably improved HER performance. The variation in the salt precursor concentration during the synthesis of a material is a simple methodology to produce a scalable platinum-free catalyst for HER. The advantageous features of the multiple active sites of cobalt in the CN-21 sample as compared to that for pristine CoS and NiS laid the foundation for the provision of abundant active edges for HER. The composite sample produced a current density of 10 mA cm^−2^ at an overpotential of 345 mV. Also, it exhibited a Tafel value of 60 mV dec^−1^, which predominantly ensured rapid charge transfer kinetics during HER. CN-21 was highly durable and stable for 30 hours. Electrochemical impedance spectroscopy showed that the charge transfer resistance was 21.88 ohms, which further validated the HER polarization curves and Tafel results. CN-21 exhibited a double layer capacitance of 4.69 μF cm^−2^ and a significant electrochemically active surface area of 134.0 cm^2^, which again supported the robust efficiency for HER. The obtained results reveal that our developed NiCo_2_S_4_ catalyst has a high density of active edges, and it is a non-noble metal catalyst for the hydrogen evolution reaction. The present findings provide an alternative strategy and an active nonprecious material for the development of energy-related applications.

## Abstract 2

There are many challenges associated with the fabrication of efficient, inexpensive, durable and very stable nonprecious metal catalysts for the hydrogen evolution reaction (HER).

## Graphical abstract

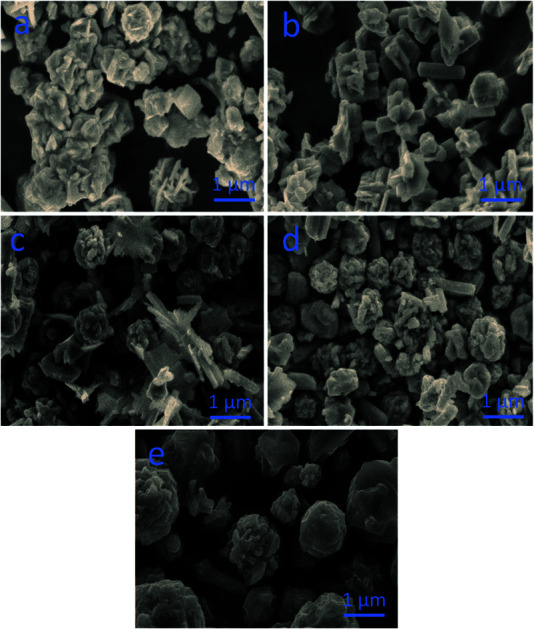

## 1. Introduction

Fossil fuels are continuously consumed, have depleting reservoirs, and contribute to global warming; hydrogen with a high density of energy has been found to be a potential energy carrier because of its renewable features, greenness, and zero carbon emission.^1–4^ Water splitting is a useful method to produce hydrogen gas on a large scale, but it is restricted by the poor performance and high cost of electrocatalysts.^5,6^ Pt-based materials are active for HER; however, the fact that platinum is a noble and scarce metal has strongly limited its scalable use.^7^ To overcome this, a wide range of transition metal substances are being investigated including metal chalcogenides, selenides, phosphides, nitrides, carbides, and metal alloys.^8–24^ Also, the attractive features of biological compounds such as nitrogenase and hydrogenase drive the development of transition metal sulfide-based HER catalysts.^25,26^ The obtained effectiveness of metal sulfides is far from that of noble metal electrocatalysts. The produced current densities show that transition metal chalcogenides are promising compounds and their tailored surface activities have great potential for the design of highly active HER catalysts in the near future. Besides this, a binary metal sulfide, namely, NiCo_2_S_4_ has good conductivity, mixed d orbitals, and unique electrochemical activities and it has been used for water oxidation/reduction reactions.^27,28^ Also, NiCo_2_S_4_ has been found to be active towards HER in alkaline media, and it produces 10 mA cm^−2^ current density at an overpotential of 210 mV.^29^ In electrochemical water splitting, electrolytes are critical to the reaction kinetics and selectivity of different phenomena including the charge transport between the electrode–electrolyte interface and the stability of the catalytic substance. They are mainly dependent on the electrolyte and its pH.^30,31^ The catalysts tend to dissolve easily under acidic conditions due to the movement of metal ions from the electrode surface.^30^ In alkaline conditions, the electrochemical processes result in the formation of metal hydroxide layers.^31^ Therefore, we changed the stoichiometry of the salts during the synthesis of bimetallic sulfide NiCo_2_S_4_ and it turned into a stable and efficient HER catalyst in acidic media. We have measured the stability at two different current densities. Importantly, this is the first report of HER using NiCo_2_S_4_ in acidic media.

In this study, we used a cost-effective approach for the synthesis of nickel–cobalt bimetallic sulfide NiCo_2_S_4_ and employed the material for HER in acidic media. The nanostructured NiCo_2_S_4_ was studied *via* a wide range of analytical methods including X-ray diffraction (XRD), high resolution transmission electron microscopy (HRTEM), and scanning electron microscopy (SEM). The catalyst exhibited 10 mA cm^−2^ current density at an overpotential of 345 mV and was found to be durable for 30 hours. Also, the charge transfer resistance, double layer capacitance, electrochemically active surface area, and exchange current density were calculated for NiCo_2_S_4_. They collectively explain the fundamental reasons for the observed efficient HER.

## 2. Materials and methods

### 2.1. Chemical reagents

Cobalt chloride hexahydrate, nickel chloride hexahydrate, thiourea, absolute ethanol, Nafion, and H_2_SO_4_ were received from Sigma Aldrich, Karachi, Pakistan.

### 2.2. Synthesis of nanostructured materials

The sulfide nanostructures were prepared using the hydrothermal method. Five solutions of pristine CoS, pristine NiS, and three NiCo_2_S_4_ composite samples with different Co : Ni ratios using different amounts of cobalt and nickel precursors were prepared by dissolving metallic chloride hexahydrate and thiourea in 70 ml of deionized water; the detailed information about the precursor concentrations is given in Table S1.† We simply optimized the ratio of cobalt concentration to the nickel concentration for the synthesis of the NiCo_2_S_4_ composite catalysts and monitored the influence of the Co : Ni ratio on the HER performance in acidic media. The Co : Ni ratios were 1 : 2, 1 : 1, and 2 : 1 for CN-12, CN-11, and CN-21, respectively, as given in Table S1.† The growth process was carried out for 12 h in a stainless-steel autoclave at 210 °C. After the growth process, the autoclave was cooled to room temperature. The nanostructured product was rinsed with distilled water and ethanol several times.

The phase and purity of the as-prepared samples were studied using a Bruker D8 Advance diffractometer operating at 40 kV and 40 mA using Cu Kα radiation (*λ* = 0.15406 nm). Quantitative Rietveld analysis was carried out to characterize the crystalline phases present using the HighScore Plus software.^32,33^ The morphology was studied by scanning electron microscopy (SEM) using a JSM-6380L JEOL scanning electron microscope. Elemental analysis was also carried out with energy dispersive spectroscopy (EDS) equipped with SEM.

All the electrochemical experiments were performed on a VERSASTAT 4-500 electrochemical workstation. A three-electrode cell set up was used, which included a glassy carbon electrode as the working electrode, a silver–silver chloride electrode (Ag/AgCl) filled with a 3 M KCl solution as the reference electrode, and a graphite rod as the counter electrode. All the measurements were performed in 0.5 M sulfuric acid as the electrolyte. The catalyst material was deposited on glassy carbon electrodes using the drop casting technique. Prior to the HER polarization curves, cyclic voltammetry (CV) was performed in order to stabilize the working electrode at 5 mV s^−1^. The polarization curves were recorded with linear sweep voltammetry (LSV) at a scan rate of 5 mV s^−1^. The electrochemically active surface area was calculated from the cyclic voltammograms at different scan rates (*i.e.*, 30, 50, and 70 mV s^−1^). The durability test was examined through chronopotentiometry at 10 mA cm^−2^ and 20 mA cm^−2^ current densities over a period of 30 h. EIS measurements were performed in a frequency range of 100 kHz to 1 Hz using a sinusoidal potential of 10 mV and the HER onset potential. The experimental potentials are reported against the reversible hydrogen electrode (RHE) using the Nernst equation.

## 3. Results and discussion

### 3.1. The physical characterization of various metal sulfide nanostructures

**Fig. 1:**
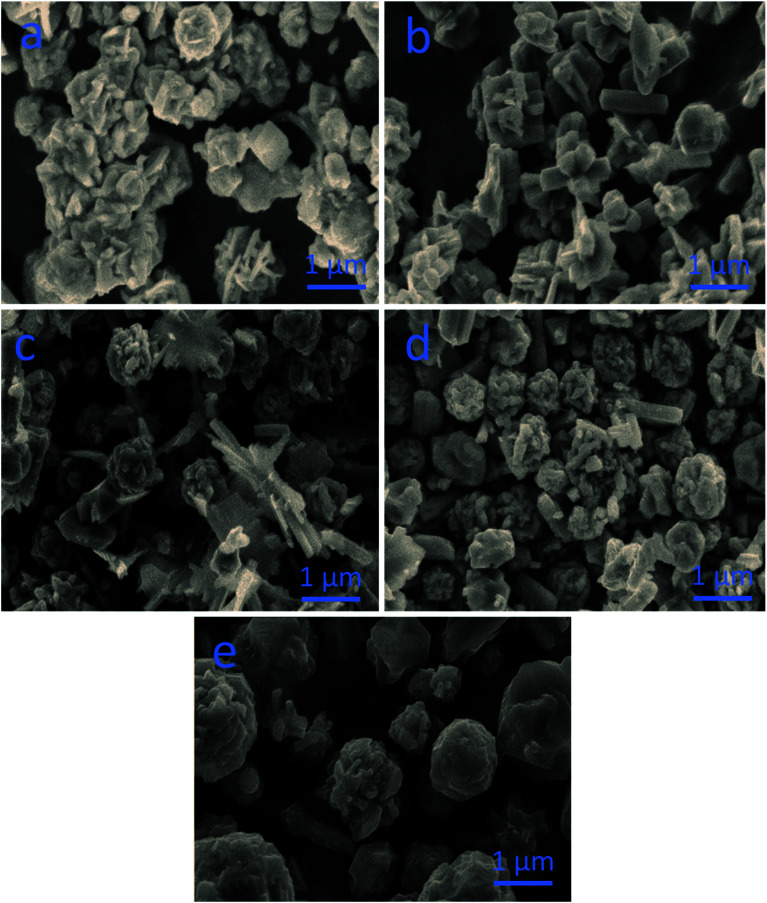
SEM images of various materials: (a) pristine cobalt sulfide, (b) pristine nickel sulfide, (c) CN-12, (d) CN-11, (e) CN-21.

The SEM images demonstrate the morphologies of the as-prepared nanostructured materials (Fig. 1). Pristine CoS comprised microparticles with a size of few microns, as shown in Fig. 1a. Pristine NiS consisted of nanorods that were randomly oriented with a length of few microns and an average diameter of 300–500 nm, as shown in Fig. 1b. CN-12 and CN-11 possessed a mixture of large microparticles and nanorods, as shown in Fig. 1c and d, respectively. CN-21 comprised a majority of microflower-like structures (Fig. 1e).

**Fig. 2:**
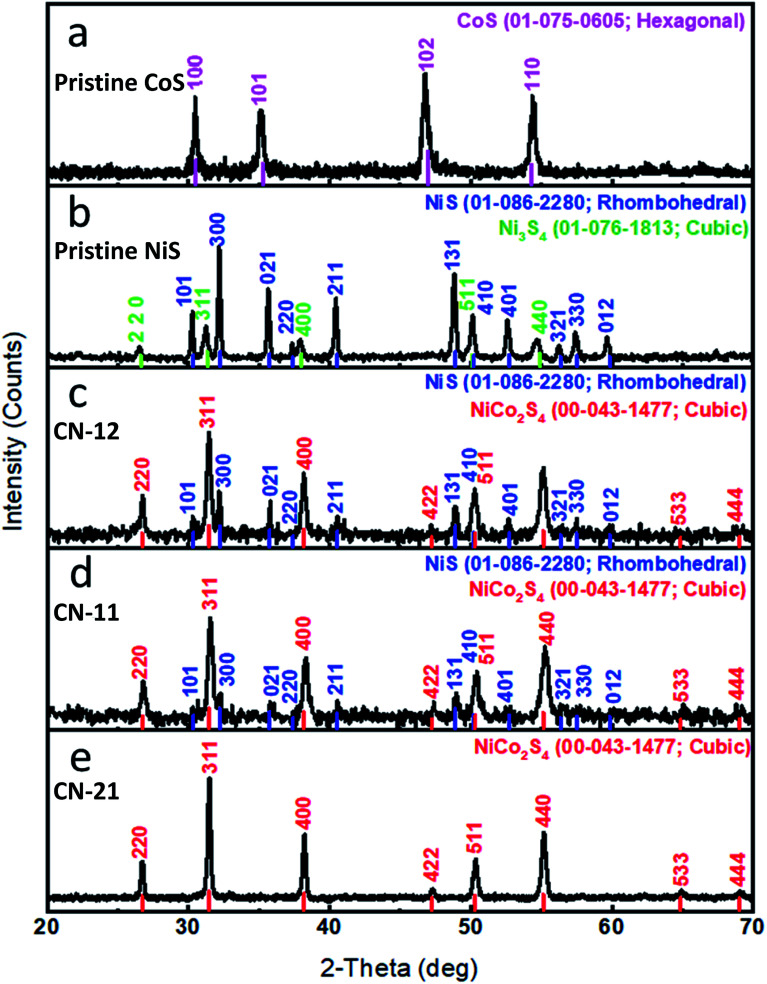
XRD patterns: (a) pristine cobalt sulfide, (b) pristine nickel sulfide, (c) CN-12, (d) CN-11, (e) CN-21.

The crystalline phases of pristine NiS and CoS were investigated by powder XRD, as shown in Fig. 2. All the diffraction patterns of pristine CoS were in good agreement with the reference JCPDS card no. = 01-075-0605 and it exhibited a hexagonal phase. Pristine NiS exhibited a rhombohedral phase and its diffraction patterns well matched to the reference card no. 01-086-2280. However, it demonstrated few reflection peaks from the cubic Ni_3_S_4_ nanostructured material and they completely agreed with the reference card no. 01-076-1813. The composite sample was identified as NiCo_2_S_4_ and its diffraction patterns were in full agreement with the standard card no. 00-043-1477 with cubic crystallography. The diffraction peaks of Ni_3_S_4_ and NiCo_2_S_4_ are very close to each other, as shown in Fig. 2. In the case of pristine nickel sulfide, the presence of Ni_3_S_4_ can be confirmed by the diffraction peaks at the 2-theta values of 26.638, 31.346, 38.03, 50.078, and 54.871, corresponding to the 220, 311, 400, 511, and 440 planes. However, for the other samples, the diffraction peaks at the 2-theta values of 26.744, 31.475, 38.195, 47.245, 50.304, 55.126, 64.878, and 69.031 correspond to the 220, 311, 400, 422, 511, 440, 533, and 444 planes, confirming the presence of the NiCo_2_S_4_ phase. The XRD study of CN-21 revealed the successful preparation of the NiCo_2_S_4_ nanostructure. No other impurity phase was detected through XRD analysis.

The chemical composition of various samples was obtained using the HighScore Plus Software (Quantitative Rietveld analysis) and is shown in Table S2.† This confirmed the increased cobalt percentage, which efficiently enhanced the HER activity.

**Fig. 3:**
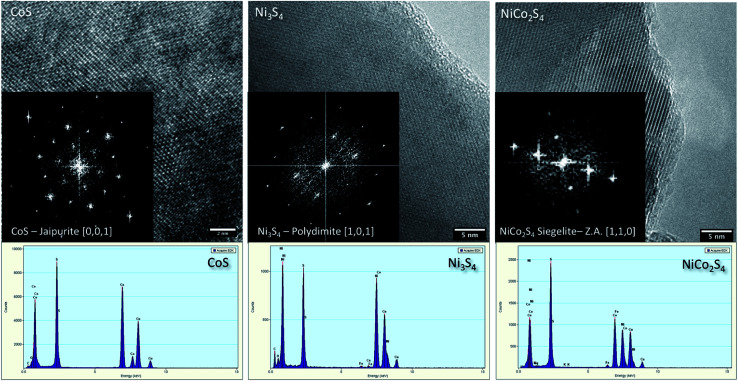
High resolution TEM micrographs from left pristine cobalt sulfide, middle pristine nickel sulfide and right NiCo_2_S_4_ (CN-21); insets show the corresponding FFT patterns. In the bottom parts of the images, the EDX spectra of the relative samples are displayed.

Fig. 3 exhibits the HR-TEM characterization for the prepared samples, specifically for pristine CoS, NiS, and CN-21. The high-magnification images confirm the highly crystalline nature of the materials and the reflection patterns can be successfully indexed to the same phases identified by XRD. The EDX spectra confirm the presence of Co, S, Ni, and S, and Co, Ni, and S are the main elements in pristine CoS, pristine NiS, and NiCo_2_S_4_ samples, respectively. The EDX mapping of CN-21 (Fig. S1†) also confirmed the homogeneous distribution of cobalt, nickel, and sulfur even though there was a slight variation in the Ni/Co ratios, which suggested a more complex structure. However, it must be noted that no variation in the crystal lattice was observed within each nanostructure, indicating that the slight variations in the local composition did not affect the crystal structure.

### 3.2. HER measurements in acidic media

**Fig. 4:**
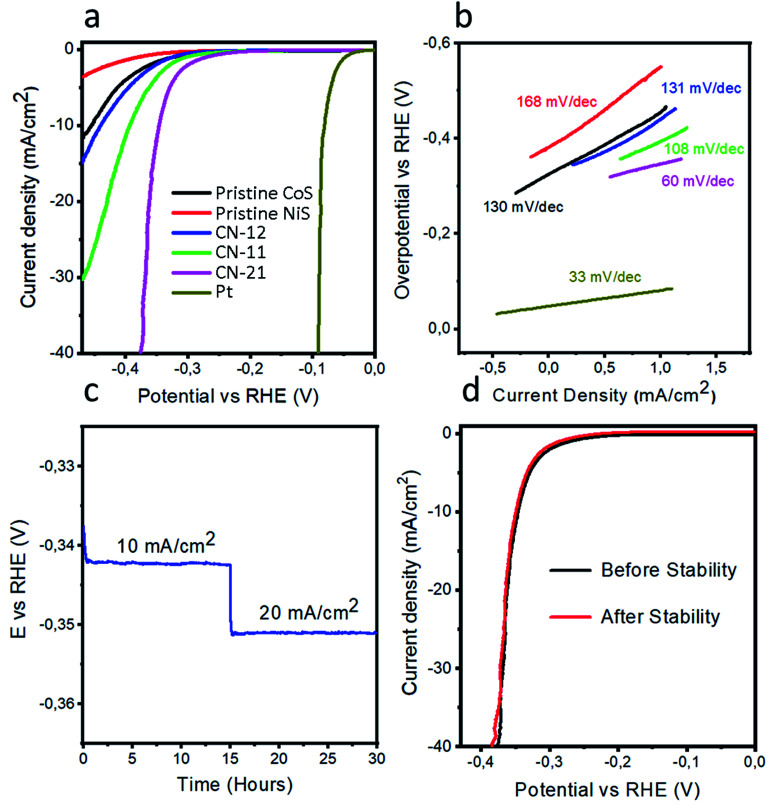
(a) Polarization curves for HER at 5 mV s^−1^ in 0.5 M H_2_SO_4_ solution; (b) extracted Tafel plots from the HER polarization curves; (c) chronoamperometric durability test for CN-21 for 30 hours at various current densities in 0.5 M H_2_SO_4_; (d) stability results of CN-21 (NiCo_2_S_4_) before and after the chronoamperometry test.

We measured the HER performance and kinetics of pristine CoS, pristine NiS, and various NiCo_2_S_4_ composite catalysts by LSV using a three-electrode configuration, as depicted in Fig. 4a. For better understanding, the HER activity of the nonprecious metal catalysts was differentiated with 20 wt% Pt/C. An excellent HER performance was demonstrated by the noble Pt catalyst, which was expected. The composite samples exhibited enhanced HER activity when compared to pristine CoS and NiS. For the composite samples, the variation in the precursor concentration of either cobalt chloride or nickel chloride hexahydrate altered the HER performance. Therefore, optimization in terms of the precursor salt concentration was carried out and CN-21 showed excellent HER activity, which indicated that an increase in the concentration of the cobalt precursor in the composite had a prominent effect on the functional properties. CN-21 exhibited a low overpotential of 345 mV, while CN-11, CN-12, pristine CoS, and pristine NiS demonstrated large overpotentials. A 10 mA cm^−2^ current density was measured at 345 mV, which was either equal or superior to that of many active noble metal-free catalysts (Table S3†). Furthermore, it was observed that the variation in the precursor salt, especially cobalt chloride, could significantly alter the phases of the nanostructures, which reduced the overpotential, as in the case of CN-21. This improved HER performance can be ascribed to the synergetic effect produced in CN-21, which has better conductivity, as illustrated by the LSV polarization curves. For CN-21, it is probable that most of the active sites originate from cobalt and nickel, which can enhance the HER activity. Generally, Tafel analysis is performed to describe the electron transfer kinetics of a catalyst, which is related to and originates from the HER kinetics of the catalyst.^34^ The Tafel plots are calculated from the Tafel equation, as indicated in Fig. 4b. Interestingly, CN-21 displayed a Tafel value of 60 mV dec^−1^, which was smaller than that of pristine CoS (130 mV dec^−1^), NiS (168 mV dec^−1^), CN-12 (131 mV dec^−1^), and CN-11 (108 mV dec^−1^). This analysis revealed that CN-21 was accompanied by the superior HER kinetics of charge transport and rapid ion diffusion during water dissociation, which was in close agreement with the LSV result. CN-21 exhibited a Tafel value of 60 mV dec^−1^, which confirmed the faster HER kinetics and electrochemical desorption determined the rate of reaction.^35^ Another parameter to understand the HER process is exchange current density (Table 1); CN-21 exhibited the highest exchange current density of 1.61 × 10^−3^ A cm^−2^ when compared to pristine CoS, pristine NiS, CN-12, and CN-11. The high exchange current density of CN-21 indicates its efficient HER kinetics. The superior performance of CN-21 could be assigned to the single phase (NiCo_2_S_4_), which might reduce the hydrogen adsorption energy. However, for CN-12 and CN-11, the nickel content showed slight improvement in the HER activity. The optimized Co to Ni ratio has shown the presence of NiS in the CN-12 and CN-11 and thier performance is not up to mark performance when compared to the CN-21. Therefore, a higher cobalt to nickel ratio is favorable for HER activity and it can also be attributed to the pure phase of NiCo_2_S_4_ as in CN-21.

**Table 1:** Summary of the unique features of the presented HER catalysts

Catalyst	Calculated from LSV		Calculated from EIS		Calculated from CV	
	Tafel slope	Exchange current density	Charge transfer resistance	Double layer capacitance	Double layer capacitance	Electrochemically active surface area
	*B* (mV dec^−1^)	*m* (A cm^−2^)	*R*_ct_ (Ω)	CPE_dl_ (mF)	*C*_dl_ (μF cm^−2^)	ECSA (cm^2^)
Pristine cobalt sulfide	130	2.82 × 10^−4^	54.62	0.63	1.79	51.1
Pristine nickel sulfide	168	8.14 × 10^−5^	125.4	0.33	1.37	39.1
CN-12	131	3.76 × 10^−4^	49.92	0.43	1.91	54.6
CN-11	108	4.89 × 10^−4^	33.57	0.73	3.11	88.9
CN-21	60	1.61 × 10^−3^	21.88	0.97	4.69	134.0

Chronopotentiometry experiments were carried out on CN-21 using the current densities of 10 mA cm^−2^ and 20 mA cm^−2^ for 15 h each, as shown in Fig. 4c. These measurements revealed a negligible loss of overpotential; this indicated that CN-21 exhibited excellent durability and could be used for long term applications without the loss of HER activity. The stability of nonprecious metal catalysts is another parameter to evaluate the HER performance and it was determined before and after the durability measurements using the LSV curves, as depicted in Fig. 4d. It was found that CN-21 was highly stable and retained its HER onset potential and current density.

**Fig. 5:**
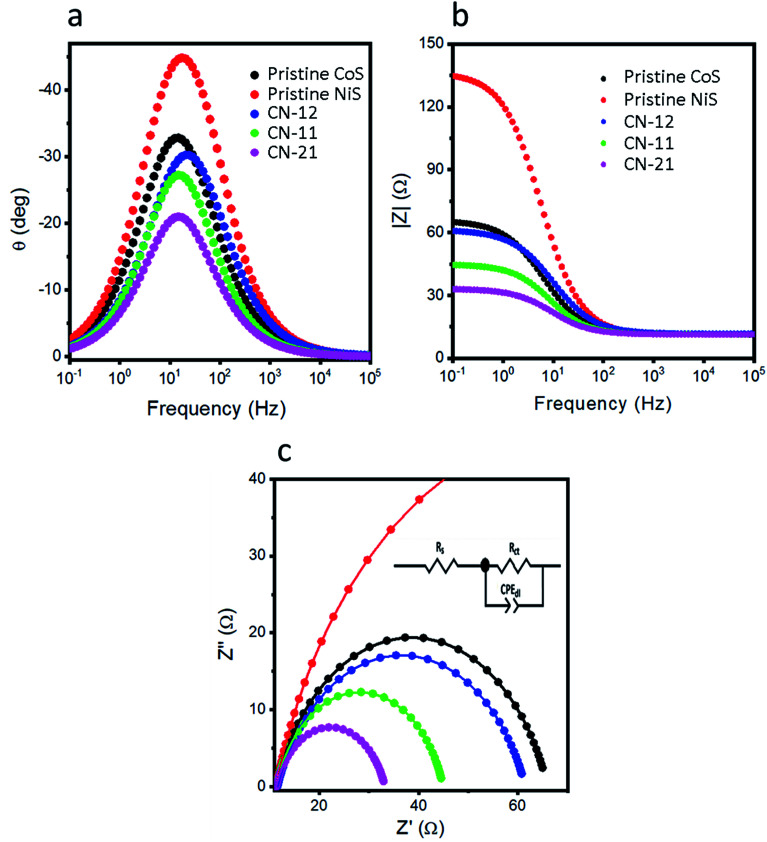
EIS experiments in 0.5 M H_2_SO_4_ from 100 kHz to 0.1 Hz and bias potential of 10 mV and HER onset potential of 345 mV *vs.* RHE. (a and b) The obtained Bode plots from impedance results and (c) Nyquist plots for pristine cobalt sulfide, pristine nickel sulfide, CN-12, CN-11, and CN-21.

Electrochemical impedance spectroscopy (EIS) is another possible path to measure the ionic transport and charge transfer rate of catalysts in the HER process (Fig. 5). The Nyquist plots are shown in Fig. 5a and the inset indicates the corresponding equivalent circuit. The Bode plots, shown in Fig. 5b and c, were measured from the same impedance results. The straightforward hint we can get from EIS suggests that a smaller charge transfer resistance indicates fast charge transfer kinetics.^36^ The charge transfer resistance values of pristine CoS, pristine NiS, CN-12, CN-11, and CN-21 were 54.6, 125.4, 49.9, 33.56, and 21.9 ohms, respectively. This analysis confirmed that CN-21 exhibited low charge transfer resistance with boosted HER performance. EIS indicated that the variation in the precursor concentration during the synthesis of NiCo_2_S_4_ altered the conductivity of CN-12, CN-11, and CN-21. Clearly, a higher amount of the cobalt precursor results in rapid ion migration and consequently superior HER performance, as demonstrated in this study. From the EIS study, we also quantified the capacitance values for different sulfide materials and CN-21 exhibited a higher capacitance value of 0.97 mF, which is another supporting parameter for explaining the robust HER performance. The electrochemically active surface area (ECSA) was quantified from the CV curves at various scan rates, as shown in S1.† Generally, it is believed that the value of *C*_dl_ is linearly related to ECSA.^14^ Interestingly, the *C*_dl_ values of pristine CoS, pristine NiS, CN-12, CN-11, and CN-21 were 1.79, 1.37, 1.91, 3.11, and 4.69 μF cm^−2^, respectively, as shown in Fig. S2f.† The ECSA value was higher for CN-21, *i.e.*, approximately 134.0 cm^−2^, as supported by the higher *C*_dl_ value. This further confirms that CN-21 has a high density of catalytic active sites due to the higher amount of cobalt in CN-21, which strengthens the superior HER activity. The obtained values of *C*_dl_ and ECSA from the CV experiments are also listed in Table 1.

## 4. Conclusions

In summary, we have produced a composite sample of NiCo_2_S_4_ by tailoring the precursor concentrations using the hydrothermal method. SEM characterization showed that a mixture of microparticles and nanorods was present in the composite sample. XRD and HRTEM analyses confirmed the presence of a cubic phase with excellent crystallinity. Through the exploitation of bimetallic active sites with optimum ratios, we found an enhancement in the HER activity in acidic media. A 10 mA cm^−2^ current density was achieved at 345 mV for the sample containing a higher amount of cobalt. A Tafel value of 60 mV dec^−1^ revealed faster HER kinetics, and excellent 30 hour stability was observed. A small charge transfer resistance and high double-layer capacitance were noted, suggesting that the synergistic effect of the decreased charge transfer resistance and the increased number of active edges resulted in optimal catalytic activity. Therefore, this study provides a further understanding of the properties of mixed Ni : Co sulfides with respect to HER, opening the way for the design of novel, highly active catalytic substances for HER and diverse applications.

## Conflicts of interest

Authors declare no conflict of interest in this research work.

## Supplementary Material

RA-010-D0RA03191G-s001
