# Supplementary material for: Nickel–cobalt bimetallic sulfide NiCo2S4 nanostructures for a robust hydrogen evolution reaction in acidic media
Source: RSC Adv. 2020 Jun 15;10(37):22196–203. doi: 10.1039/d0ra03191g (PMC9054539; doi:10.1039/d0ra03191g)
Supplement: RA-010-D0RA03191G-s001 [file RA-010-D0RA03191G-s001.pdf]

Electronic Supplementary Material (ESI) for RSC Advances.
This journal is © The Royal Society of Chemistry 2020

SUPPORTING INFORMATION (SI)

Nickel cobalt bimetallic sulfide $NiCo_2S_4$ nanostructures for robust hydrogen evolution reaction in alkaline media

Umair Aftab*[a], Aneela Tahira[b], Raffaello Mazzaro[c], Vittorio Morandi[c], Muhammad Ishaq Abro[a], Muhammad Moazam Baloch[a], Cong Yu[d], Zafar Hussain Ibupoto*[e]

[a]Mehran University of Engineering and Technology, 7680 Jamshoro, Sindh Pakistan

[b]Department of Science and Technology, Campus Norrkoping, Linkoping University, SE-60174 Norrkoping, Sweden

[c]☐Institute for Microelectronics and Microsystems, Italian National Research Council, Section of Bologna, Via Piero Gobetti 101, 40129, Bologna, Italy.

[d]State Key Laboratory of Electroanalytical Chemistry, Changchun Institute of Applied Chemistry Chinese Academy of Sciences Changchun People's Republic of China

[e]Dr. M.A Kazi Institute of Chemistry University of Sindh Jamshoro, 76080, Sindh Pakistan

***Corresponding author:** Zafar Hussain Ibupoto, PhD and Umair Aftab

Email address: zaffar.ibhupoto@usindh.edu.pk, umair.aftab@faculty.muet.edu.pk

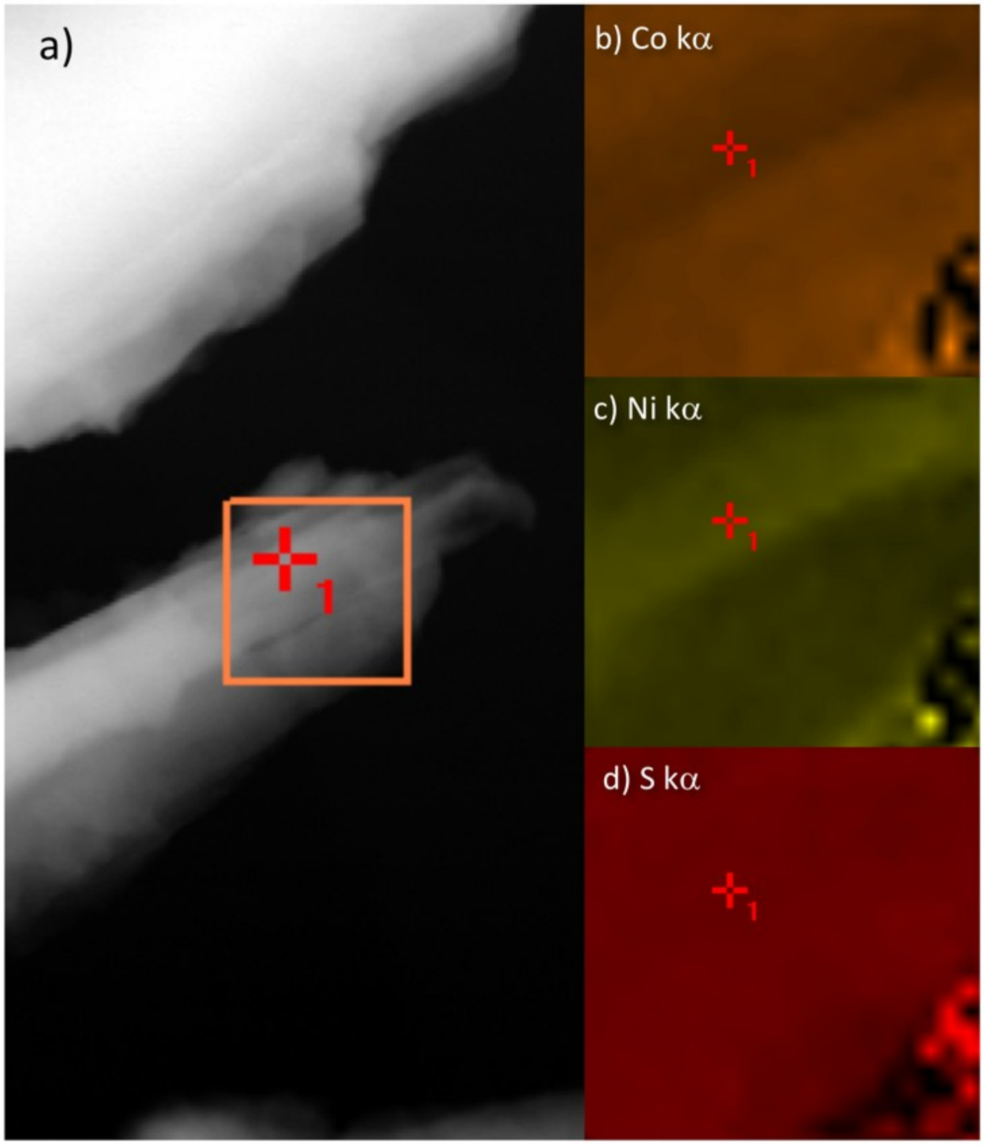

Figure S1: a) STEM-HAADF micrograph of the tip of a $NiCo_2S_4$ crystal and relative EDX distribution map for b) Co, c) Ni and d) S content.

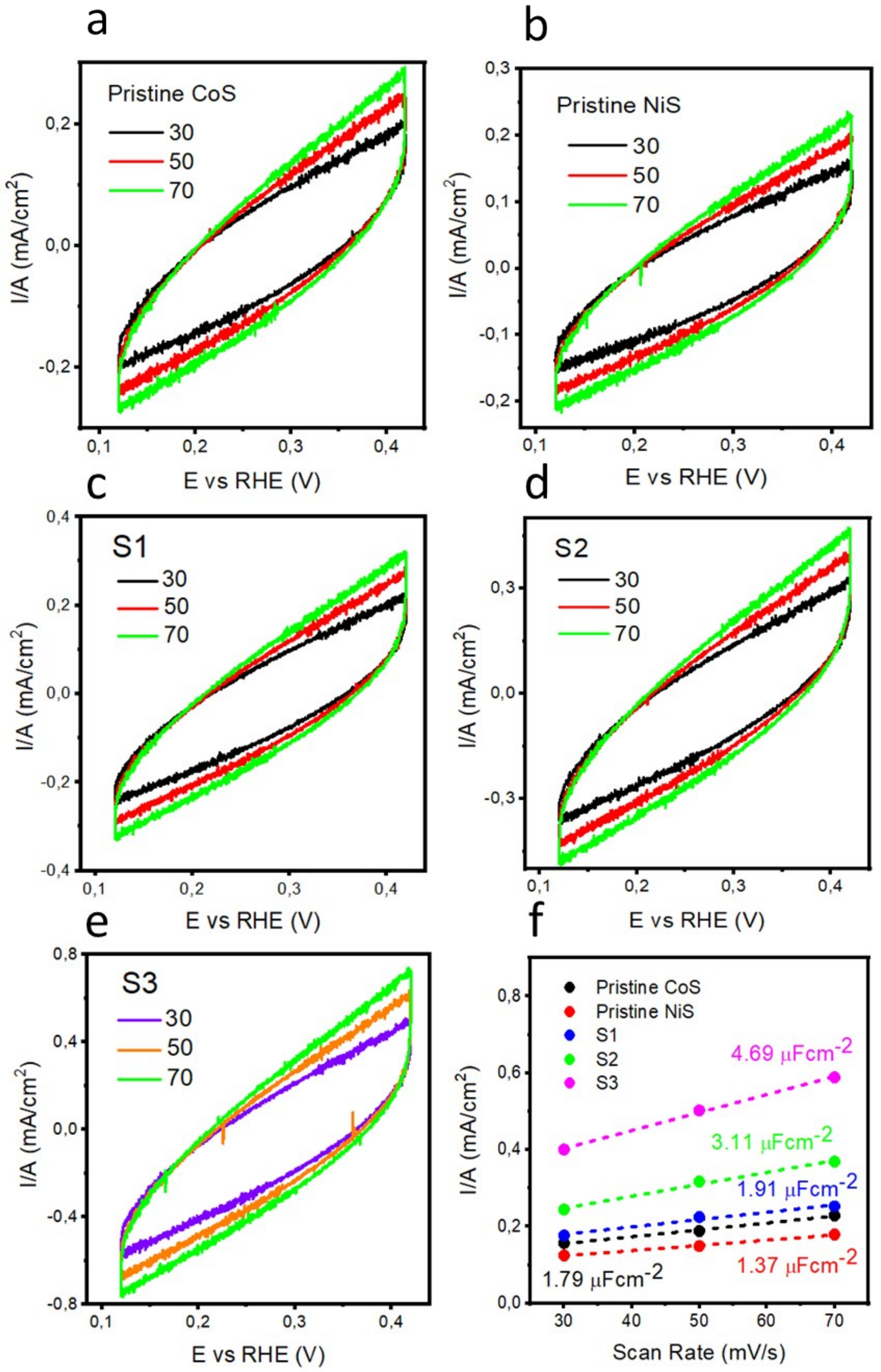
a
Pristine CoS
30
50
70
I/A (mA/cm^2)
E vs RHE (V)
b
Pristine NiS
c
S1
d
S2
e
S3
f
Pristine CoS
Pristine NiS
S1
S2
S3
4.69 μFcm^{-2}
3.11 μFcm^{-2}
1.91 μFcm^{-2}
1.79 μFcm^{-2}
1.37 μFcm^{-2}
Scan Rate (mV/s)

Figure S2: CV curves of various materials at different scan rates (a) pristine cobalt sulfide, (b) pristine nickel sulfide, (c) CN-12, (d) CN-11, (e) CN-21, (f) Linear fitting of average current density vs scan rate for the calculation of electrochemical active surface area

Table S1: Sample ID and their recipe

Sample ID	Cobalt chloride hexahydrate	Nickel chloride hexahydrate	Thiourea	Deionized Water
**Pristine cobalt sulfide**	666mg	---	532mg	70ml
**Pristine nickel sulfide**	---	666mg	532mg	70ml
**CN-12**	222mg	444mg	532mg	70ml
**CN-11**	333mg	333mg	532mg	70ml
**CN-21**	444mg	222mg	532mg	70ml

Table S2: Composition analysis using XRD

Sample ID	Co	Ni	S
**Pristine cobalt sulfide**	64.81%	0.00%	35.19%
**Pristine nickel sulfide**	0.00%	63.35%	36.65%
**CN-12**	19.35%	38.54%	42.11%
**CN-11**	29.02%	28.91%	42.07%
**CN-21**	38.70%	19.27%	42.03%

Table S3: Electrochemical HER comparison of previously reported catalysts.

Catalyst	Electrolyte	Potential vs RHE (mV)	Current Density (mA cm^{-2})	Tafel Slope (mVdec^{-1})	Reference
Cu-MoS_2/rGO	0.5M H_2SO_4	-400	83.6	90	[1]
Mo_2N-MoS_2	0.5M H_2SO_4	-400	175	59	[2]
N-CNF	0.5M H_2SO_4	-490	10	154	[3]
WSe_2-CFM	0.5M H_2SO_4	-295	30	80	[4]
WSe_2-CFP	0.5M H_2SO_4	-300	10	77.4	[5]
$NiCo_2S_4$	0.5 M H_2SO_4	-345	10	60	This work

References:

1. F. Li, L. Zhang, J. Li, X. Lin, X. Li, Y. Fang, J. Huang, W. Li, M. Tian, J. Jin, R. Li, *J Power Sources*, 2015, **292**, 15–22.
2. K. Ojha, S. Saha, S. Banerjee, A.K. Ganguli, *ACS Appl Mater Interfaces*, 2017, **9**, 19455–19461.
3. D.F. Niu, Y. Ding, Z.X. Ma, M.H. Wang, Z. Liu, B.W. Zhang, X.S. Zhang, *Acta Chim. Sinica*, 2015, **73**, 729–734.

4. M. Zou, J. Zhang, H. Zhu, M. Du, Q. Wang, M. Zhang, X. Zhang, *J. Mater. Chem. A*, 2015, **3**, 12149–12153.
5. H. Wang, D. Kong, P. Johanes, J.J. Cha, G. Zheng, K. Yan, N. Liu, Y. Cui, *Nano Lett.*, 2013, **13**, 3426–3433.
